# Predictive Value of the CALLY Index for Interventional Management in Vaginal Cuff Hematoma Following Hysterectomy

**DOI:** 10.3390/diagnostics16101561

**Published:** 2026-05-21

**Authors:** Candost Hanedan, Ayşe Nur İnal, Ayşe Yiğit, Oğuz Kaan Köksal, Şahin Kaan Baydemir, Neslihan Öztürk, Hande Nur Öncü, Gökçen Ege, Aysu Yeşim Tezcan, Tuba Zengin Aksel, Vakkas Korkmaz, Çağanay Soysal

**Affiliations:** 1 Department of Gynecologic Oncology, Ankara Etlik City Hospital, 06790 Ankara, Turkey; 2 Department of Obstetrics and Gynecology, Ankara Etlik City Hospital, 06790 Ankara, Turkey; 3 Department of Obstetrics and Gynecology, Lokman Hekim University, 06790 Ankara, Turkey

**Keywords:** vaginal cuff hematoma, CALLY index, hysterectomy, inflammatory biomarkers, postoperative complication, risk stratification, neutrophil-to-lymphocyte ratio, systemic immune-inflammation index

## Abstract

**Background/Objectives:** Vaginal cuff hematoma is a recognized complication following hysterectomy, with a subset of patients requiring invasive intervention. No reliable bedside biomarker currently exists to identify at admission patients likely to fail conservative management. This study aimed to evaluate the incidence and clinical characteristics of symptomatic vaginal cuff hematoma across all hysterectomy approaches, and to assess the predictive performance of the CALLY index (CRP-albumin-lymphocyte index), a composite marker of inflammatory burden, immune function, and nutritional status, alongside the neutrophil-to-lymphocyte ratio (NLR), platelet-to-lymphocyte ratio (PLR), and systemic immune-inflammation index (SII) for identifying patients requiring interventional management. **Methods:** This retrospective cohort study included 61 patients with symptomatic vaginal cuff hematoma following hysterectomy in a major tertiary referral center (November 2022–July 2025). Patients were divided into conservative (n = 38) and interventional (n = 23) management groups. The CALLY index was calculated as [Albumin (g/dL) × Lymphocyte (×10^9^/L)] ÷ [CRP (mg/L) × 10^−2^]. Receiver operating characteristic (ROC) curve analysis with the DeLong method was used to compare predictive performance. **Results:** The overall incidence of symptomatic vaginal cuff hematoma was 1.9% (73/3852 hysterectomies), with the highest rate following vaginal hysterectomy (3.32%) and the lowest after robotic hysterectomy (0.74%). Interventional management was required in 37.7% of patients. The interventional group had significantly higher CRP (192 vs. 62 mg/L, *p* < 0.001), NLR (7.53 vs. 4.17, *p* < 0.001), and SII (2308 vs. 1207, *p* < 0.001), and significantly lower CALLY index values (2.00 vs. 9.80, *p* < 0.001). The CALLY index demonstrated the highest predictive performance (AUC = 0.863, 95% CI: 0.762–0.964), outperforming SII (AUC = 0.801), NLR (AUC = 0.789), and PLR (AUC = 0.654). At the optimal cutoff of ≤2.89, the CALLY index yielded a sensitivity of 65.2% and a specificity of 92.1%. **Conclusions:** The CALLY index is a simple, routinely available composite biomarker that may help identify patients at higher risk for interventional management in symptomatic vaginal cuff hematoma. Its incorporation into postoperative assessment may improve risk stratification and support timely clinical decision-making.

## 1. Introduction

Hysterectomy is one of the most frequently performed gynecological procedures worldwide and is commonly indicated for benign conditions such as symptomatic leiomyomas, abnormal uterine bleeding, adenomyosis, and uterine prolapse [1,2]. Despite advancements in minimally invasive surgery, including laparoscopic, robotic, and vaginal Natural Orifice Transluminal Endoscopic Surgery (v-NOTES) approaches, postoperative complications remain a recognized concern following hysterectomy, with reported rates ranging from 5% to 20% depending on the surgical approach and patient characteristics [3,4]. Among these, vaginal cuff hematoma is a relatively common but underreported complication that may range from incidental sonographic findings to clinically significant conditions requiring intervention [5,6].

Previous studies have reported a higher risk of pelvic or cuff hematomas following vaginal hysterectomy compared to abdominal approaches [6,7]. Although most cases are managed conservatively, the vaginal route has been identified as a potential risk factor for infected hematomas [6]. Most vaginal cuff hematomas resolve with conservative measures such as observation or antibiotic therapy; however, in a proportion of cases, progression to severe symptoms or secondary infection necessitates surgical evacuation or additional interventional procedures, including transvaginal catheter drainage and repeat surgical intervention (laparoscopy or laparotomy), leading to prolonged hospitalization and delayed recovery [8,9].

Determining reliable early predictors of the need for intervention is therefore clinically essential. In this context, systemic inflammatory and immune-nutritional indices have gained attention as candidate tools for risk stratification. The CALLY index, formulated from C-reactive protein (CRP), albumin and lymphocyte count has emerged as a composite biomarker reflecting inflammatory burden, immune function, and nutritional status, with its prognostic value increasingly recognized across multiple cancer types, including gynecologic malignancies [10,11]. While its utility in oncologic settings is well established, its role in benign gynecologic postoperative complications has not been explored. In parallel, inflammatory indices derived from routine complete blood count, including the neutrophil-to-lymphocyte ratio (NLR), platelet-to-lymphocyte ratio (PLR), and systemic immune-inflammation index (SII), have been evaluated as predictors of surgical intervention in gynecologic pelvic infections, with conflicting results [12,13]. However, their applicability to vaginal cuff hematoma following hysterectomy remains unclear. Incorporating such indices may provide a novel approach to stratify patients with vaginal cuff hematoma according to their likelihood of requiring additional intervention.

The aim of this study was to evaluate the incidence, clinical characteristics, and treatment outcomes of vaginal cuff hematoma following hysterectomy for gynecologic indications in a major tertiary referral center. Specifically, we compared patients managed conservatively with those requiring additional interventional procedures, including transvaginal catheter drainage, repeat surgical intervention (laparoscopy), or laparotomy, and assessed the predictive performance of the CALLY index, NLR, PLR, and SII for identifying patients likely to require such interventions.

## 2. Materials and Methods

### 2.1. Study Design and Patients

**Figure 1:**
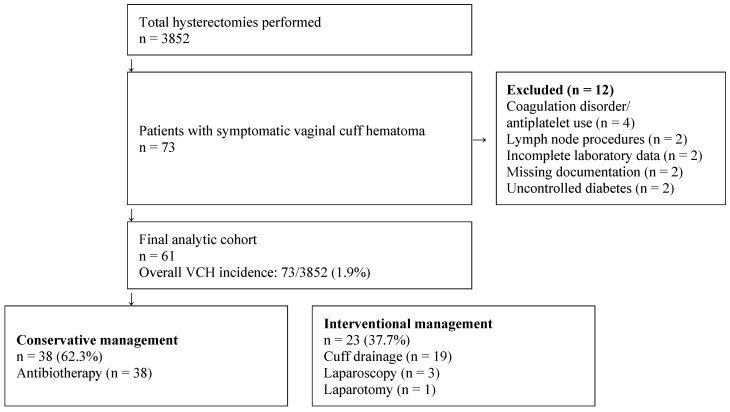
Patient selection flowchart. VCH: vaginal cuff hematoma.

This retrospective cohort study was conducted at the Department of Gynecology and Gynecologic Oncology, Etlik City Hospital, a major tertiary referral center, and included patients treated between November 2022 and July 2025. The study received ethical approval from the Institutional Review Board (AEŞH-BADEK2-2025-516; 25 August 2025). Data were retrospectively collected from medical records of patients treated during the study period. Although retrospective in design, all patients had provided written informed consent at the time of their index surgery as part of standard preoperative institutional practice, in accordance with institutional regulations and consistent with the principles of the Declaration of Helsinki. The patient selection process is illustrated in Figure 1. The final analytic cohort comprised 61 patients with symptomatic vaginal cuff hematoma.

### 2.2. Inclusion and Exclusion Criteria

Patients were eligible for inclusion if they had undergone hysterectomy without concomitant oncologic staging procedures and subsequently presented with symptomatic vaginal cuff hematoma confirmed by ultrasonography. Symptomatic vaginal cuff hematoma was defined as an ultrasonographically confirmed hematoma associated with at least one of the following clinical findings: pelvic pain, fever (temperature ≥38.0 °C on two measurements at least 30 min apart), vaginal bleeding, or foul-smelling vaginal discharge, necessitating hospital admission [6,7,8]. Exclusion criteria were as follows: (i) obstetric-related hematomas; (ii) hematologic or coagulation disorders or current use of anticoagulant or antiplatelet agents; (iii) concomitant oncologic or retroperitoneal lymph node procedures; (iv) uncontrolled diabetes mellitus; (v) incomplete laboratory data; and (vi) missing clinical documentation. The patient selection process and exclusion criteria are detailed in Figure 1.

### 2.3. Study Groups

Patients were divided into two groups based on clinical management: the conservative group (n = 38), comprising patients managed with antibiotic therapy with or without additional supportive measures; and the interventional group (n = 23), comprising patients who required additional procedural intervention directly targeting the hematoma, including transvaginal catheter drainage and repeat surgical intervention (laparoscopy or laparotomy).

### 2.4. Surgical Procedures

All hysterectomies were performed by surgeons experienced in gynecologic surgery at a major tertiary referral center. Surgical approaches included abdominal, laparoscopic, vaginal, robotic, and vNOTES hysterectomy, and were selected based on uterine characteristics, patient comorbidities, and clinical judgment. All procedures were performed under general or regional anesthesia.

Perioperative care was conducted in accordance with enhanced recovery after surgery (ERAS) principles, including standard antibiotic prophylaxis administered within 60 min before skin incision and vaginal antiseptic preparation with povidone-iodine solution prior to hysterectomy. Perioperative antibiotic prophylaxis consisted of intravenous cefazolin 1 g and metronidazole 500 mg administered within 60 min before skin incision. In cases where operative time exceeded 3 h, a repeat dose of cefazolin was administered intraoperatively in accordance with standard pharmacokinetic principles. Venous thromboembolism (VTE) prophylaxis was administered according to the Caprini risk assessment model, patients with lower risk scores received short-term prophylaxis, whereas those with higher risk scores received extended low-molecular-weight heparin prophylaxis. Nasogastric tubes and abdominal drains were not routinely used. For vaginal hysterectomy procedures, including vNOTES, a vaginal pack was routinely placed at the end of surgery and removed approximately 4 h after the procedure.

### 2.5. Management of Vaginal Cuff Hematoma

The incidence of vaginal cuff hematoma was calculated for each hysterectomy approach and is presented in Table 1. Vaginal cuff hematoma was defined as a hypoechoic or heterogeneous collection adjacent to the vaginal cuff, identified on ultrasonography as the primary imaging modality, with computed tomography or magnetic resonance imaging performed when clinically indicated [14]. Infected hematoma was defined as a hematoma associated with fever (≥38.0 °C on two occasions at least 30 min apart), pelvic pain, or foul-smelling vaginal discharge, or cases requiring surgical drainage, in the absence of an alternative diagnosis [6,7,8].

Upon presentation, all patients underwent standardized microbiological evaluation including urine, blood, and vaginal cultures. In patients who underwent interventional management, additional microbiological cultures were obtained from the hematoma cavity at the time of drainage. Conservative management consisted of standardized intravenous antibiotic therapy, comprising ceftriaxone 1 g twice daily plus metronidazole 500 mg three times daily, or gentamicin plus clindamycin when indicated, administered for 5–7 days, followed by oral therapy when appropriate, along with analgesia, hydration, and bed rest. Antibiotic regimens were modified in cases of inadequate clinical response, defined as persistent or rising CRP levels in conjunction with clinical deterioration, including worsening pelvic pain, persistent fever, or hemodynamic instability, or when culture results demonstrated resistance to the empirical regimen.

Interventional management included transvaginal catheter drainage and repeat surgical intervention (laparoscopy or laparotomy), performed based on clinical judgment and hematoma characteristics including size, symptoms, and clinical course. Patients undergoing vaginal cuff drainage underwent transvaginal cuff re-exploration under anesthesia, with hematoma evacuation, saline irrigation, hemostasis by bipolar cautery or sutures, and re-approximation of the cuff using delayed-absorbable sutures. In cases where transvaginal access was insufficient or hematoma characteristics precluded this approach, repeat surgical intervention (laparoscopy) or laparotomy was performed, with hematoma evacuation, irrigation, and hemostasis as indicated. When multiple measurements were available, values closest to the time of hematoma diagnosis were used. CRP was measured using a standard immunoturbidimetric assay; the institutional upper limit of normal was defined as 10 mg/L. A significant decline in CRP was defined as a substantial decrease from admission values, irrespective of whether strict normalization below 10 mg/L was achieved.

The CALLY index, NLR, PLR, and SII were retrospectively calculated for all patients using laboratory values obtained at the time of hospital admission for vaginal cuff hematoma. The CALLY index was calculated using the following formula, consistent with the methodology described by Wang et al. [10]:

CALLY = [Albumin (g/dL) × Lymphocyte (×10^9^/L)] ÷ [CRP (mg/L) × 10^−2^]

The NLR was calculated as the neutrophil count divided by the lymphocyte count, the PLR as the platelet count divided by the lymphocyte count, and the SII as the product of the neutrophil count and platelet count divided by the lymphocyte count (SII = Neutrophil × Platelet ÷ Lymphocyte).

### 2.6. Statistical Analysis

Statistical analyses were performed using SPSS software (version 31.0.2.0, IBM Corp., Armonk, NY, USA). Given the retrospective nature of this study, no formal a priori sample size calculation was performed. The Shapiro–Wilk test was used to assess the normality of continuous variables. Since all continuous variables were non-normally distributed, they are expressed as median (interquartile range). Categorical variables are expressed as frequencies and percentages. Differences between the conservative and interventional management groups were assessed using the Mann–Whitney U test for continuous variables and the chi-square or Fisher’s exact test for categorical variables, as appropriate.

Paired comparisons of laboratory parameters between admission and discharge were performed using the Wilcoxon signed-rank test. Receiver operating characteristic (ROC) curve analysis was performed to evaluate the predictive performance of the CALLY index, NLR, PLR, and SII for identifying patients requiring interventional management. Area under the curve (AUC) values with 95% confidence intervals were calculated using the DeLong method. Optimal cutoff values were determined using the Youden index (sensitivity + specificity − 1). Sensitivity, specificity, positive predictive value (PPV), and negative predictive value (NPV) were calculated for each index at the optimal cutoff. Statistical significance was defined as *p* < 0.05.

## 3. Results

### 3.1. Incidence and Patient Characteristics

A total of 3852 hysterectomies were performed during the study period, of which 73 patients were readmitted with symptomatic vaginal cuff hematoma, corresponding to an overall incidence of 1.9%. Following application of exclusion criteria, 61 patients were included in the final analysis (Figure 1). The incidence by surgical approach was 2.18% after abdominal hysterectomy, 1.56% after laparoscopic, 3.32% after vaginal, 0.74% after robotic, and 0.99% after vNOTES hysterectomy (Table 1). The highest incidence was observed following vaginal hysterectomy, whereas the lowest was after robotic hysterectomy. Among the study population, only one patient had an incidental postoperative diagnosis of endometrial cancer following robotic hysterectomy performed without oncologic staging procedures; all remaining cases were benign.

### 3.2. Demographic and Clinical Characteristics

The median age of the cohort was 49 years (IQR 46–58), and the median BMI was 28 kg/m^2^ (IQR 24–32). The most common indication for hysterectomy was uterine fibroid (n = 17, 27.9%), followed by endometrial intraepithelial neoplasia (n = 15, 24.6%), abnormal uterine bleeding (n = 11, 18.0%), uterine prolapse (n = 9, 14.8%), adnexal mass (n = 6, 9.8%), CIN (cervical intraepithelial neoplasia) (n = 2, 3.3%), and other indications (n = 1, 1.6%). Regarding surgical approach, abdominal and laparoscopic hysterectomy were equally the most common routes (n = 21 each, 34.4%), followed by vaginal (n = 12, 19.7%), vNOTES (n = 6, 9.8%), and robotic hysterectomy (n = 1, 1.6%). The median postoperative presentation day was 10 (IQR 8–12), and the median length of hospital stay was 6 days (IQR 4–8). Prior abdominal surgery was reported in 22 patients (36.1%), including prior cesarean section in 14 (23.0%). Demographic and clinical characteristics of the study population are summarized in Table 2.

### 3.3. Conservative vs. Interventional Management Group Comparison

Of the 61 patients, 38 (62.3%) were managed conservatively and 23 (37.7%) required interventional management. Among patients requiring intervention, 19 underwent transvaginal cuff drainage, three underwent laparoscopic evaluation/intervention (including one patient who also underwent vaginal cuff drainage), and one required laparotomy. Patients in the interventional group had significantly higher BMI (30.0, IQR 27.0–35.3 vs. 27.5, IQR 24.3–31.0; *p* = 0.016) and larger hematoma size at presentation (75 mm, IQR 55–100 vs. 60 mm, IQR 46–71; *p* = 0.017). The length of hospital stay was significantly longer in the interventional group (8 days, IQR 6–11 vs. 6 days, IQR 4–7; *p* = 0.009). No significant differences were observed between groups with respect to age, parity, menopausal status, or surgical approach. Detailed comparisons are presented in Table 3.

Regarding laboratory parameters, CRP was markedly higher in the interventional group (192 mg/L, IQR 119–237 vs. 62 mg/L, IQR 30–101; *p* < 0.001), as were WBC (12.68 × 10^9^/L, IQR 10.10–16.49 vs. 10.00 × 10^9^/L, IQR 8.00–12.31; *p* = 0.007) and NLR (7.53, IQR 5.31–9.71 vs. 4.17, IQR 3.18–4.90; *p* < 0.001). Among the 23 patients who underwent interventional management, microbiological cultures were obtained at the time of drainage, with positive results identified in 13 cases (56.5%). The most frequently isolated microorganisms were *Escherichia coli* (*E. coli*) and *Enterococcus faecalis*, identified at similar frequencies. Among interventional patients, the median CALLY index was lower in culture-positive patients (1.79) compared with culture-negative patients (2.36). Albumin was significantly lower in the interventional group (3.15 g/dL, IQR 2.75–3.71 vs. 3.50 g/dL, IQR 3.20–3.92; *p* = 0.020). PLR (248, IQR 145–301 vs. 182, IQR 140–216; *p* = 0.045) and SII (2308, IQR 1738–3879 vs. 1207, IQR 932–1669; *p* < 0.001) were also significantly higher in the interventional group. The CALLY index was significantly lower in the interventional group (2.00, IQR 1.58–4.73 vs. 9.80, IQR 6.26–18.50; *p* < 0.001).

### 3.4. Laboratory Findings

**Figure 2:**
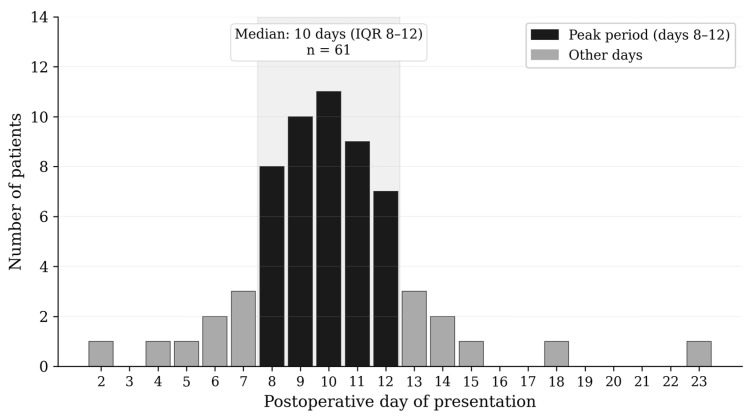
Distribution of postoperative presentation day among patients with symptomatic vaginal cuff hematoma (n = 61).

At admission, patients demonstrated a marked systemic inflammatory response, with a median CRP of 93.0 mg/L (IQR 44.0–192.5). By discharge, CRP had decreased significantly to 11.0 mg/L (IQR 5.27–20.42; *p* < 0.001). WBC counts declined from 11.30 × 10^9^/L (IQR 8.80–14.00) at admission to 7.33 × 10^9^/L (IQR 6.00–9.00) at discharge (*p* < 0.001). Body temperature normalized from 36.8 °C (IQR 36.4–37.2) to 36.4 °C (IQR 36.3–36.6; *p* < 0.001). Platelet counts did not differ significantly between admission and discharge (325 × 10^9^/L, IQR 256–378 vs. 336 × 10^9^/L, IQR 266–420; *p* = 0.255). The median time to significant CRP decline was 10 days (IQR 7–12), corresponding with the peak hematoma presentation period(Figure 2). These findings are summarized in Table 4.

### 3.5. ROC Analysis and Predictive Performance of Inflammatory Indices

**Figure 3:**
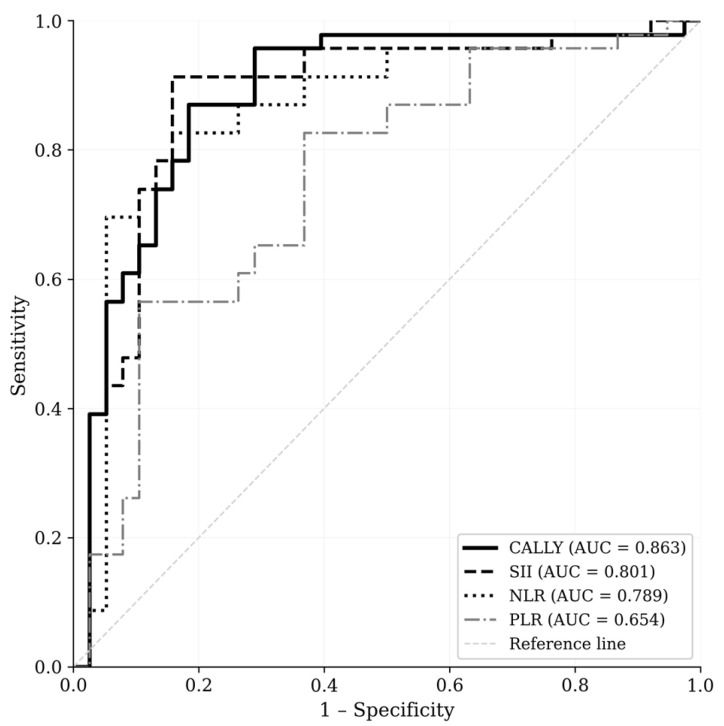
ROC curves for CALLY index, SII, NLR, and PLR for predicting interventional management in symptomatic vaginal cuff hematoma. AUC: area under the curve; CALLY: CRP-albumin-lymphocyte index; NLR: neutrophil-to-lymphocyte ratio; PLR: platelet-to-lymphocyte ratio; SII: systemic immune-inflammation index.

ROC curve analysis was performed to evaluate the predictive performance of the CALLY index, NLR, PLR, and SII for identifying patients requiring interventional management (Figure 3). The CALLY index demonstrated the highest discriminatory ability, with an AUC of 0.863 (95% CI: 0.762–0.964; *p* < 0.001). At the optimal cutoff of ≤2.89, the CALLY index yielded a sensitivity of 65.2%, specificity of 92.1%, PPV of 83.3%, and NPV of 81.4%.

The SII demonstrated an AUC of 0.801 (95% CI: 0.680–0.922; *p* < 0.001), with an optimal cutoff of ≥1813, yielding a sensitivity of 73.9% and specificity of 78.9%. The NLR had an AUC of 0.789 (95% CI: 0.664–0.914; *p* < 0.001), with an optimal cutoff of ≥5.73, sensitivity of 73.9%, and specificity of 86.8%. The PLR demonstrated the lowest discriminatory ability among the four indices, with an AUC of 0.654 (95% CI: 0.509–0.799; *p* = 0.045), optimal cutoff of ≥238, sensitivity of 56.5%, and specificity of 89.5%.

The CALLY index outperformed all other indices in terms of AUC. These findings are summarized in Table 5.

## 4. Discussion

In this retrospective cohort study, we investigated the incidence, clinical characteristics, and management outcomes of symptomatic vaginal cuff hematoma following hysterectomy, with particular focus on the predictive performance of the CALLY index and other systemic inflammatory indices, NLR, PLR, and SII, for identifying patients requiring interventional management. To our knowledge, this is the first study to evaluate the CALLY index in the context of a benign gynecologic postoperative complication.

The overall incidence of symptomatic vaginal cuff hematoma in our cohort was 1.9%, consistent with previously reported rates ranging from 0.7% to 2.5% [6,15,16]. The highest incidence was observed following vaginal hysterectomy (3.32%), while the lowest was after robotic hysterectomy (0.74%), consistent with prior reports suggesting that the vaginal route carries a relatively higher risk of cuff-related complications, attributed to direct cuff dissection and immediate closure predisposing to inadequate hemostasis [7,16]. The elevated incidence following vaginal hysterectomy in our cohort may be partly attributable to the clinical profile of patients undergoing this approach; vaginal hysterectomy was predominantly performed for uterine prolapse in postmenopausal women, who frequently required concomitant pelvic floor reconstructive procedures such as anterior and posterior colporrhaphy and sacrospinous ligament fixation. These additional surgical steps involve extensive vaginal dissection and prolonged operative time, which may increase the risk of inadequate hemostasis and subsequent hematoma formation, consistent with prior reports linking concomitant pelvic reconstructive procedures to higher rates of postoperative pelvic hematoma [7,8]. Notably, vNOTES hysterectomy demonstrated an incidence of 0.99%, comparable to laparoscopic and robotic approaches, suggesting that this technique does not confer additional hematoma risk despite its transvaginal nature [17]. These observations emphasize that vaginal cuff hematoma may occur regardless of surgical route, supporting the rationale for selective postoperative imaging and close clinical follow-up in high-risk patients. The standardized perioperative antibiotic prophylaxis protocol applied in our cohort may have contributed to limiting infectious complications associated with vaginal cuff hematoma.

Regarding management outcomes, 38 patients (62.3%) were successfully treated with conservative measures, while 23 (37.7%) required interventional management. This rate is consistent with previously reported series: Carter et al. described a surgical management rate of approximately 20–30% [9], and Mohapatra et al. reported that larger or infected hematomas frequently necessitate drainage procedures [7]. Among patients who underwent interventional management, microbiological cultures obtained at the time of drainage yielded positive results in 13 cases (56.5%), consistent with previously reported bacterial growth rates of 47–57% in drained infected pelvic hematomas following hysterectomy [6,8]. It is important to note that the indication for interventional management in our cohort was not exclusively infectious in nature. Among the 23 patients requiring intervention, 13 (56.5%) had positive cultures suggesting an infectious etiology, while the remaining 10 (43.5%) were culture-negative, indicating that intervention was driven primarily by mechanical factors such as hematoma enlargement, persistent symptoms, or inadequate response to conservative therapy in these cases. This distinction is clinically relevant, as the CALLY index reflects both infectious burden, through elevated CRP and suppressed albumin and lymphocyte levels, and the systemic inflammatory response associated with mechanical hematoma progression. Furthermore, the lower CALLY index values observed in culture-positive patients compared with culture-negative interventional patients (median 1.79 vs. 2.36) may support the biological plausibility of the CALLY index as a potential reflector of both inflammatory and infectious burden, although the limited sample size precluded formal statistical comparison between these subgroups. Our interventional rate of 37.7% falls within the broader range reported in the literature, and may partly reflect our strict definition of interventional management, which encompassed not only surgical drainage but also transvaginal catheter drainage and repeat surgical intervention (laparoscopy or laparotomy). Patients in the interventional group presented with significantly larger hematomas (75 mm vs. 60 mm; *p* = 0.017), higher BMI (*p* = 0.016), and longer hospital stays (8 vs. 6 days; *p* = 0.009), suggesting that hematoma size and patient-related factors may serve as early clinical indicators of the need for intervention. These findings are consistent with prior observations linking larger hematoma dimensions and obesity to increased risk of infectious complications and treatment failure [6,16].

At hospital admission, patients demonstrated a marked systemic inflammatory response, reflected by significantly elevated CRP levels (median 93.0 mg/L). By discharge, CRP had declined significantly to 11.0 mg/L (*p* < 0.001), with a median time to significant CRP decline of 10 days (IQR 7–12), corresponding with the peak hematoma presentation period. Although discharge CRP levels approached but did not consistently fall below the institutional upper limit of normal (10 mg/L) in all patients, the magnitude of decline was clinically meaningful in both groups. This pattern is consistent with prior studies demonstrating that CRP serves as a sensitive and dynamic marker of postoperative infectious complications following hysterectomy [18,19]. WBC counts and body temperature were similarly normalized by discharge, further corroborating clinical resolution. Platelet counts, however, remained relatively stable throughout the clinical course, with no significant difference between admission and discharge values (*p* = 0.255), suggesting that platelet levels were not substantially altered by the inflammatory process associated with vaginal cuff hematoma in this cohort [6,16].

When comparing groups, CRP was markedly higher in the interventional group (192 vs. 62 mg/L; *p* < 0.001), as were WBC and NLR, while albumin was significantly lower. These findings suggest that the degree of systemic inflammatory response at presentation may reflect the severity of the underlying hematoma and its infectious burden, thereby influencing the likelihood of requiring procedural intervention. However, CRP alone, due to its nonspecific nature, may not adequately discriminate between patients who will respond to conservative therapy and those requiring intervention, underscoring the need for composite inflammatory indices such as the CALLY index [18,19].

To our knowledge, this is the first study to evaluate the CALLY index in the setting of a benign gynecologic postoperative complication. The CALLY index, calculated as albumin multiplied by lymphocyte count divided by CRP, demonstrated the strongest predictive performance among all indices evaluated, with an AUC of 0.863 (95% CI: 0.762–0.964). At the optimal cutoff of ≤2.89, it yielded a sensitivity of 65.2% and a specificity of 92.1%, indicating that a CALLY index above this threshold effectively identifies patients likely to respond to conservative management. The biological rationale for this finding is compelling: in patients with severe hematoma-associated inflammation, CRP rises markedly while albumin and lymphocyte counts decline, reflecting a state of heightened inflammatory burden, impaired nutritional status, and suppressed cellular immunity, all of which may contribute to hematoma progression and the failure of conservative treatment [10,11]. In clinical practice, the CALLY index may be used at admission to stratify patients according to their risk of requiring intervention, allowing for more tailored management strategies and timely escalation of care. In exploratory multivariable logistic regression analysis (Table 6), BMI and hematoma size remained independently associated with the need for interventional management, whereas the association between the CALLY index and interventional management attenuated after adjustment and demonstrated borderline statistical significance. These findings suggest that although the CALLY index showed the strongest discriminatory performance in ROC analysis, part of its predictive ability may reflect the overall inflammatory burden and disease severity associated with larger hematomas and higher BMI. Nevertheless, the consistently lower CALLY index values observed in the interventional group support its potential utility as an adjunctive biomarker for early risk stratification.

Inflammatory indices were evaluated using laboratory parameters obtained at hospital admission in order to assess their potential utility for early risk stratification. Dynamic longitudinal changes in these biomarkers during hospitalization were not systematically analyzed and may provide additional prognostic information in future prospective studies.

Among the other inflammatory indices evaluated, the SII demonstrated the second highest discriminatory ability (AUC = 0.801), followed by NLR (AUC = 0.789) and PLR (AUC = 0.654). Both SII and NLR showed acceptable predictive performance, with sensitivities of 73.9% for both indices, suggesting their potential utility as complementary screening tools when CALLY index calculation is not readily available.

The predictive value of NLR and SII in gynecologic infectious and inflammatory conditions has been previously reported by Kose et al. and Peker et al., albeit with conflicting results [12,13]. In the context of tubo-ovarian abscess and pelvic inflammatory disease, Kose et al. demonstrated that elevated SII was associated with treatment failure and need for surgical intervention, while Peker et al. similarly reported significant associations between NLR, PLR, SII and medical treatment response, consistent with our findings [12,13]. However, to our knowledge, neither NLR, PLR, nor SII has been previously evaluated as a predictor of interventional management specifically in vaginal cuff hematoma, rendering direct comparison with prior literature limited.

The PLR demonstrated the weakest predictive performance in our cohort (AUC = 0.654), which may reflect the inherent variability of platelet counts in the acute postoperative setting, where platelet count variability in the acute postoperative period may confound its discriminatory ability [6,16]. This finding suggests that platelet-based indices may be less reliable in this specific clinical context compared to indices incorporating CRP and albumin, such as the CALLY index.

The strengths of this study include the systematic evaluation of vaginal cuff hematoma across all major hysterectomy approaches, the novel application of the CALLY index in a benign gynecologic setting, and the standardized perioperative management protocol. The inclusion of multiple inflammatory indices allowed direct comparative analysis of their predictive performance, providing a comprehensive framework for clinical decision-making.

This study has several limitations that warrant consideration. First, the retrospective single-center design may limit the generalizability of our findings. Twelve patients were excluded due to various criteria, representing 16.4% of the initially identified cohort; although this proportion is relatively small, selection bias cannot be entirely excluded, particularly with respect to laboratory-based indices such as the CALLY index. Second, the relatively small sample size, especially in the interventional group (n = 23), may have limited the statistical power for secondary outcomes such as PLR. Third, the vaginal cuff closure technique was not standardized across surgical approaches; the potential impact of suture type on hematoma formation rates remains unclear and warrants further investigation, as demonstrated by Uccella et al., who reported significantly higher cuff hematoma rates following transvaginal compared to laparoscopic closure [20]. Fourth, as a retrospective study, unmeasured confounders such as operative time, intraoperative blood loss, and individual surgical technique variability could not be fully accounted for. Furthermore, the small number of patients in the major intervention subgroup (laparoscopy n = 3, laparotomy n = 1) precluded meaningful statistical comparison between minor and major intervention types; future prospective studies should stratify intervention types a priori to better characterize predictive thresholds across different intervention categories.

In conclusion, symptomatic vaginal cuff hematoma following hysterectomy is a recognized postoperative complication, with an overall incidence of 1.9% in our cohort and an interventional management rate of 37.7%. The CALLY index demonstrated the strongest predictive performance for identifying patients requiring interventional management, outperforming NLR, PLR, and SII, with an AUC of 0.863 and a specificity of 92.1% at the optimal cutoff of ≤2.89. These findings suggest that the CALLY index may serve as a simple, readily available, and clinically useful biomarker for early risk stratification in patients presenting with symptomatic vaginal cuff hematoma. Incorporation of this index into routine postoperative assessment may facilitate timely clinical decision-making and optimize resource utilization. Prospective multicenter studies with larger sample sizes are warranted to validate these findings and establish standardized cutoff values across diverse patient populations and surgical settings.

## Figures and Tables

**Table 1:** Incidence of vaginal cuff hematoma by hysterectomy type.

Hysterectomy Type	Total Procedures (n)	Hematoma (n)	Incidence (%)
Abdominal (AH)	1425	31	2.18
Laparoscopic (LH)	1727	27	1.56
Vaginal (VH)	361	12	3.32
Robotic (RH)	136	1	0.74
vNOTES	203	2	0.99
Total	3852	73	1.9

AH: abdominal hysterectomy; LH: laparoscopic hysterectomy; VH: vaginal hysterectomy; RH: robotic hysterectomy; vNOTES: vaginal natural orifice transluminal endoscopic surgery.

**Table 2:** Demographic and clinical characteristics of patients.

Variables	n = 61
Age (years), median (IQR)	49 (46–58)
BMI (kg/m^2^), median (IQR)	28 (24–32)
Parity, median (IQR)	2 (0–6)
Smoking, n (%)	3 (4.9)
Menopause, n (%)	24 (39.3)
Comorbidities, n (%)	
None	25 (41.0)
Diabetes mellitus	9 (14.8)
Hypertension	16 (26.2)
Others ^†^	11 (18.0)
Hematoma size at presentation (mm), median (IQR)	64 (50–80)
Postoperative presentation day, median (IQR)	10 (8–12)
Length of hospital stay (days), median (IQR)	6 (4–8)
Prior abdominal surgery, n (%)	22 (36.1)
Prior cesarean section, n (%)	14 (23.0)
**Indication for hysterectomy, n (%)**	
Abnormal Uterine Bleeding (AUB)	11 (18.0)
Uterine fibroid	17 (27.9)
Uterine prolapse	9 (14.8)
Adnexal mass	6 (9.8)
CIN	2 (3.3)
EIN	15 (24.6)
Others ^†^	1 (1.6)
**Type of primary surgery, n (%)**	
Abdominal	25 (41.0)
Laparoscopic	22 (36.1)
Vaginal	11 (18.0)
Robotic	1 (1.6)
vNOTES	2 (3.3)
**Management, n (%)**	
Conservative (observation/antibiotherapy)	38 (62.3)
Cuff drainage (vaginal)	19 (31.1)
Laparoscopy *	3 (4.9)
Laparotomy	1 (1.6)

^†^ Others include patients with comorbidities such as hypothyroidism and hypercholesterolemia. AUB: abnormal uterine bleeding; EIN: endometrial intraepithelial neoplasia; CIN: cervical intraepithelial neoplasia; BMI: body mass index; IQR: interquartile range; vNOTES: vaginal natural orifice transluminal endoscopic surgery. * Data are presented for the final analytic cohort (n = 61) following exclusion of 12 patients; therefore, the surgical type of distribution differs from Table 1, which reflects the total identified hematoma cohort (n = 73) prior to exclusions. One patient in the interventional group underwent both transvaginal cuff drainage and laparoscopic evaluation.

**Table 3:** Comparison of clinical and laboratory parameters: conservative vs. interventional management.

Parameter	Conservative (n = 38) Median (IQR)	Intervention (n = 23) Median (IQR)	*p*-Value
Clinical parameters			
Age (years)	49 (46–58)	50 (48–62)	0.347
BMI (kg/m^2^)	27.5 (24.3–31.0)	30.0 (27.0–35.3)	0.016
Hematoma size (mm)	60 (46–71)	75 (55–100)	0.017
Presentation day	9 (8–11)	10 (7–15)	0.281
Hospital stay (days)	6 (4–7)	8 (6–11)	0.009
Standard laboratory parameters			
CRP (mg/L)	62 (30–101)	192 (119–237)	<0.001
WBC (×10^9^/L)	10.00 (8.00–12.31)	12.68 (10.10–16.49)	0.007
Platelet (×10^9^/L)	314 (257–341)	331 (252–466)	0.301
Albumin (g/dL)	3.50 (3.20–3.92)	3.15 (2.75–3.71)	0.020
Neutrophil (×10^9^/L)	7.80 (5.46–10.00)	10.21 (8.84–12.86)	0.002
Lymphocyte (×10^9^/L)	1.81 (1.45–2.25)	1.49 (1.00–1.88)	0.028
Inflammatory indices			
NLR	4.17 (3.18–4.90)	7.53 (5.31–9.71)	<0.001
PLR	182 (140–216)	248 (145–301)	0.045
SII	1207 (932–1669)	2308 (1738–3879)	<0.001
CALLY index	9.80 (6.26–18.50)	2.00 (1.58–4.73)	<0.001

Data are presented as median (IQR). Mann–Whitney U test was used for all comparisons. NLR: neutrophil-to-lymphocyte ratio; PLR: platelet-to-lymphocyte ratio; SII: systemic immune-inflammation index; CALLY: CRP-albumin-lymphocyte index.

**Table 4:** Laboratory parameters at admission and discharge.

Laboratory Parameter	Admission Median (IQR)	Discharge Median (IQR)	*p*-Value
CRP (mg/L)	93.0 (44.0–192.5)	11.0 (5.27–20.42)	<0.001
WBC (×10^9^/L)	11.30 (8.80–14.00)	7.33 (6.00–9.00)	<0.001
Hemoglobin (g/dL)	10.8 (9.4–11.6)	10.4 (9.1–11.4)	0.124
Platelet count (×10^9^/L)	325 (256–378)	336 (266–420)	0.255
Body temperature (°C)	36.8 (36.4–37.2)	36.4 (36.3–36.6)	<0.001
Time to significant CRP decline (days)	—	10 (7–12)	—

Data are presented as median (IQR). Wilcoxon signed-rank test was used for paired comparisons. CRP: C-reactive protein; WBC: white blood cell.

**Table 5:** ROC analysis: predictive performance of inflammatory indices.

Index	AUC (95% CI)	Optimal Cutoff	Sensitivity % (95% CI)	Specificity % (95% CI)	PPV %	NPV %	*p*-Value
CALLY	0.863 (0.762–0.964)	≤2.89	65.2 (44.9–81.2)	92.1 (79.2–97.3)	83.3	81.4	<0.001
SII	0.801 (0.680–0.922)	≥1813	73.9 (53.5–87.5)	78.9 (63.7–88.9)	68.0	83.3	<0.001
NLR	0.789 (0.664–0.914)	≥5.73	73.9 (53.5–87.5)	86.8 (72.7–94.2)	77.3	84.6	<0.001
PLR	0.654 (0.509–0.799)	≥238	56.5 (36.8–74.4)	89.5 (75.9–95.8)	76.5	77.3	0.045

AUC values calculated by DeLong method. Optimal cutoff determined by Youden index. PPV: positive predictive value; NPV: negative predictive value.

**Table 6:** Exploratory multivariable binary logistic regression analysis: independent predictors of interventional management in vaginal cuff hematoma.

Variable	OR	95% CI	*p*-Value
BMI (kg/m^2^)	1.206	1.044–1.393	**0.011**
Hematoma size at admission (mm)	1.044	1.012–1.077	**0.007**
CALLY index	0.946	0.892–1.002	0.060

OR: odds ratio; CI: confidence interval. Bold *p*-values indicate statistical significance (*p* < 0.05). Model fit: Nagelkerke R^2^ = 0.274; overall model *p* < 0.001. Outcome: need for interventional management (yes/no).

## Data Availability

The data presented in this study are not publicly available due to patient privacy regulations and institutional data governance policies. De-identified data may be made available upon reasonable request to the corresponding author, subject to approval by the Institutional Ethics Committee.
